# Hyperbaric Oxygen Therapy for Carbon Monoxide-Induced Delayed Neuropsychiatric Sequelae: Case Report of Two Cases and Relevant Literature Review

**DOI:** 10.1155/2021/6663824

**Published:** 2021-03-02

**Authors:** Akira Monji, Hiroshi Tateishi, Toru Murakawa, Jun Matsushima, Yutaka Kunitake, Takumi Shiraishi, Ryohei Kojima, Takayoshi Inaba, Takahiro Kato, Yoshito Mizoguchi

**Affiliations:** ^1^Department of Psychiatry, Faculty of Medicine, Saga University, Saga, Japan; ^2^Department of Neuropsychiatry, Faculty of Medicine, Graduate School of Medical Sciences, Kyushu University, Japan

## Abstract

We herein report two cases with carbon monoxide- (CO-) induced delayed neuropsychiatric sequelae (DNS) successfully treated with hyperbaric oxygen therapy (HBOT) in attempt suicide by charcoal burning. The two patients with CO-induced DNS were successfully treated with a total of more than 100 sessions of HBOT. Frontal assessment battery (FAB) was useful to examine the effectiveness of HBOT objectively. In the future study, a large-randomized trial is required to establish the efficacy of HBOT for the treatment of DNS.

## 1. Introduction

Carbon monoxide (CO) is an odorless and colorless gas produced as a byproduct of incomplete combustion of carbon-based fuels and substance. The affinity of hemoglobin for CO is over 200 times of its affinity for oxygen. CO can thus displace oxygen from hemoglobin easily. CO has the toxic effect of tissue hypoxia and produces various systemic and neurological complications [1, 2]. Hyperbaric oxygen therapy (HBOT) is often recommended for patients with acute CO poisoning, especially in those with a carboxyhemoglobin concentration > 25%, coma, or cardiac dysfunction. It has been reported that three sessions of HBOT within the 24 h period of acute CO poisoning appeared to reduce the risk of cognitive sequelae after acute CO poisoning as compared with the normobaric oxygen therapy [2, 3]. Some people who survived the insult of acute CO intoxication might develop neuropsychiatric sequelae after a lucid interval from days to weeks, usually within one month. Delayed neuropsychiatric sequelae (DNS) are estimated to occur in 10 to 30 percent of victims, and it includes various symptoms such as cognitive change, parkinsonism, urinary and fecal incontinence, dementia, and psychosis. Effectiveness of HBOT for the treatment of DNS is not fully established as compared with that in the treatment for acute CO poisoning [4, 5]. Only a few studies reported a therapeutic effect of HBOT on DNS [6–10]. CO intoxication by charcoal burning is often seen as a method of suicide attempt in Japan. We herein report a case with carbon monoxide-induced DNS successfully treated with HBOT in attempt suicide by charcoal burning.

## 2. Case Report

### 2.1. Case 1

**Figure 1:**
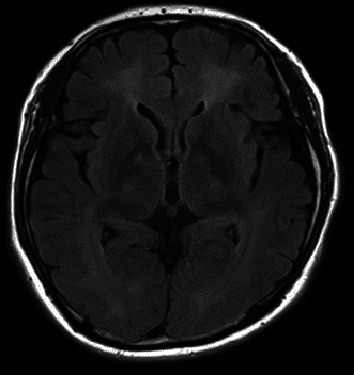
Magnetic resonance imaging (MRI) 28 days after the suicide attempt of case 1.

**Figure 2:**
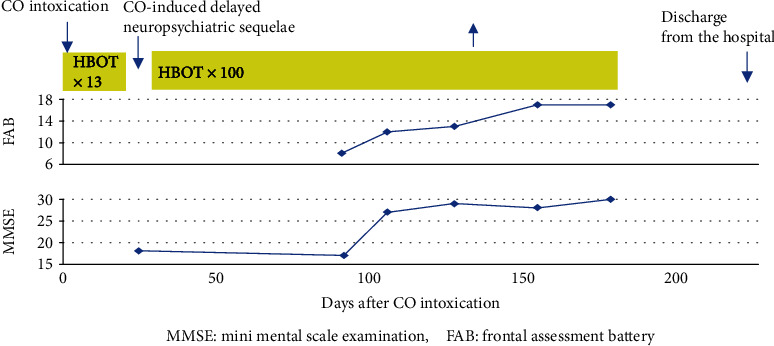
Clinical course of case 1 with neuropsychiatric assessments. CO: carbon monoxide; HBOT: hyperbaric oxygen therapy.

A 47-year-old male had attempted suicide by burning charcoal in the morning and was found unconscious that evening by his wife. He had no past history of psychiatric disorders while his younger brother had a past history of depression. He was taken to the emergency room of a local hospital where the level of carboxyhemoglobin was 38.5%. He was diagnosed as having acute CO intoxication. After 13 sessions of HBOT (2.5 atmosphere absolute (ATA) for 120 min), his consciousness retuned to the normal level. Fifteen days after his suicide attempt, he was transferred to our university hospital. Twenty-two days after his suicide attempt, his condition deteriorated with symptoms of declined cognitive functioning, aphasia, apraxia, dysphagia, muscle rigidity, and urine and fecal incontinence. He could not talk, walk, eat, and respond to any stimulation and even displayed decorticate-like posture when bedridden. He was diagnosed as having DNS of CO intoxication. Brain magnetic resonance imaging (MRI) at that time showed diffuse hyperintensity of the bilateral hemispheric white matters consistent with changes of delayed leukoencephalopathy as a result of prior CO injury with hyperintense change of the bilateral globus pallidi (Figure 1). The EEG at that time showed predominant theta (6~7 Hz) waves with poor alpha (8 Hz) waves. HBOT (2.5 ATA for 120 min) was scheduled once a day, 5 days a week, immediately after admission. After 50 sessions of HBOT, his disorientation gradually began to ameliorate. A total of 100 sessions of HBOT were performed for the treatment of DNS. At the end of HBOT, he could walk without assistance, obey command, and communicate with other people. Urine and fecal incontinence was also improved. His clinical improvement went side by side with the improvement in scores of frontal assessment battery (FAB) (Figure 2); 228 days after his suicide attempt, he was discharged from our university hospital. At that time, the score of FAB was 18/18 and the EEG was normalized with dominant alpha (11 Hz) waves. He successfully went back to work 338 days after his suicide attempt. At present time, his neuropsychiatric conditions remain unchanged about two and a half years after his suicide attempt.

### 2.2. Case 2

**Figure 3:**
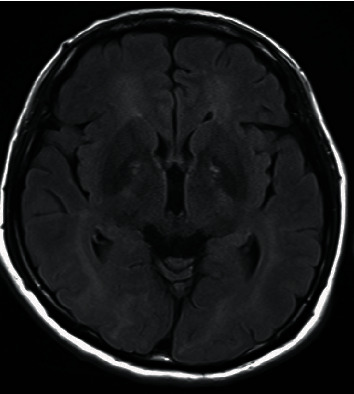
Magnetic resonance imaging (MRI) 40 days after the suicide attempt of case 2.

A 43-year-old female had attempted suicide in the morning by burning charcoal and was found unconscious in the evening by her son. She had been diagnosed as having depression for 8 years while her son had mild intellectual disability. She was taken to the emergency room of our university hospital. It was estimated that it took about 27 hours from the time of the suicide attempt to the time of admission. The level of carboxyhemoglobin was within normal range at that time. HBOT (2.5 ATA for 120 min) started immediately after admission, and her unconsciousness gradually resolved and returned to the normal level. However, after the thirteenth session of HBO, it was interrupted to treat her wound because there was a second-degree burn mainly in her upper left limb. Her disorientation and abnormal behaviors appeared about twenty days later after interruption of HBOT. She could not open the door and try to eat hand cream. The EEG at that time showed predominant slow waves (3~7 Hz theta and delta) with poor alpha (8 Hz) waves, and MRI at that time showed diffuse hyperintensity of the bilateral hemispheric white matters consistent with changes of delayed leukoencephalopathy as a result of prior CO injury with hyperintense change of the bilateral globus pallidi (Figure 3). Three sessions of HBOT were thus performed immediately. Eleven days later, she was transferred to our department for the purpose of detailed examinations and treatment of CO intoxication. Since HBOT and burn treatment could not be performed at the same time, the policy was to give priority to burn treatment. Thirty-two days later, burn treatment was finished and HBOT was thus restarted. During 136 sessions of HBOT, her neuropsychiatric symptoms gradually ameliorated. Her clinical improvement went side by side with the improvement in scores of FAB as observed in case 1. 326 days after her suicide attempt, she was discharged from our university hospital. At that time, the score of FAB was 18/18 and the EEG was normalized with dominant alpha (9 Hz) waves. At the present time, her neuropsychiatric conditions are almost well controlled for about two years and half after her suicide attempt.

## 3. Discussion

To our knowledge, there have been only a few studies on the beneficial therapeutic effect of HBOT on DNS. Myers et al. reported 10 patients with specific neurological sequelae including headaches, irritability, personality changes, confusion, and loss of memory, which developed within one to 21 days (mean, 5.7 days) after the initial CO exposure. HBOT has resulted in complete resolution of these symptoms of these ten patients [6]. Lo et al. reported 6 patients with DNS after 30-60 days of lucid interval, which was successfully treated with 8-40 sessions of HBOT [7]. Chang et al. reported 9 patients with DNS successfully treated with 8-40 sessions of HBOT [8]. Geraldo et al. reported a case with CO poisoning which, one month after the initial CO exposure, showed progressive neurobehavioral disturbance with disorientation, incoherent speech, and behavior one month. These symptoms were ameliorated with 40 sessions of HBOT [9]. Lin et al. reported a case with urine and fecal incontinence, akinetic mutism, and declined cognitive functioning due to DNS which emerged one month after the initial CO exposure. In such severe condition, the patient could regain most of his previous functions after 40 sessions of HBOT [10]. In both of the present cases, DNS emerged around twenty days after the suicide attempt and was successfully treated with more than 100 sessions of HBOT. Our present results have again demonstrated the effectiveness of HBOT for the treatment of DNS. In the present cases, we performed more sessions of HBOT than previously reported to acquire clinical improvement. FAB was very useful to examine the effectiveness of HBOT objectively in the present cases. This neuropsychiatric assessment takes less than thirty minutes to administer and is therefore practical to use in the clinical settings of DNS. Some reports have shown risk factors for DNS; however, their results have been rather inconsistent [11–14]. Therefore, as long as the clinical improvement can be detected by the neuropsychological assessment battery such as FAB, it may be desirable for us clinicians to perform HBOT therapy in as many sessions as possible. In future studies, a large-randomized trial is required to establish the efficacy of HBOT for the treatment of DNS.

Recent studies have reported that HBOT is beneficial for treating various brain disorders including ischemic brain injury and traumatic brain injury while the mechanism afforded by HBOT in the brain is poorly understood. A recent study has demonstrated that hyperoxygenation revitalizes Alzheimer's disease pathology through the upregulation of neurotrophic factors and the cognitive impairments of transgenic Alzheimer model mice. The beneficial effects of HBOT for brain disorders should thus be clarified in detail [15].

## Data Availability

The data used to support the findings of this study are available from the corresponding author upon request.
